# Emotion Recognition from Facial Expressions Considering Individual Differences in Emotional Intelligence

**DOI:** 10.3390/biomimetics11030174

**Published:** 2026-03-02

**Authors:** Yubin Kim, Ayoung Cho, Hyunwoo Lee, Mincheol Whang

**Affiliations:** 1 Department of Emotion Engineering, Sangmyung University, Seoul 03016, Republic of Korea; ubbin0127@gmail.com (Y.K.); joa6391@gmail.com (A.C.); lhw4846@naver.com (H.L.); 2 Department of Human-Centered Artificial Intelligence, Sangmyung University, Seoul 03016, Republic of Korea

**Keywords:** emotional intelligence, facial expression recognition, data consistency, affective ambiguity, data-centric emotion recognition

## Abstract

Facial expression recognition (FER) in naturalistic settings is constrained by label ambiguity and variability in stimulus–response alignment. Adopting a data-centric perspective, this study examined whether emotional intelligence (EI)-stratified training data influence FER performance by treating EI as a qualitative factor associated with affective data consistency. Naturally elicited facial expressions were collected in a controlled emotion induction experiment with subjective arousal and valence ratings. Using response-driven labeling, neutral ratings were retained as indicators of ambiguity. Participants were grouped into High and Low EI based on the alignment between subjective evaluations and outputs from a pretrained affect estimator. Identical binary classifiers for arousal and valence recognition were trained while varying only the training data composition and evaluated across baseline, unambiguous, and ambiguous test sets using independent training repetitions with repetition-level statistical aggregation. EI-stratified training was associated with statistically detectable, context-dependent performance differences: group effects were observed primarily under baseline conditions and, to a lesser extent, under ambiguous conditions, whereas no reliable differences emerged under unambiguous conditions. Pooled discrimination differences were modest, but item-level analyses identified significant differences in classification correctness in specific task–condition combinations. Comparable patterns were observed across alternative backbone architectures. These findings indicate that FER performance in naturalistic contexts is influenced not only by model architecture but also by the statistical structure and internal coherence of the training data, supporting EI-informed data selection in ambiguity-prone scenarios.

## 1. Introduction

The rapid growth of remote interactions and hyper-personalized services has increased the need for techniques that can estimate users’ momentary affective changes accurately and reliably in digital environments. Accordingly, artificial intelligence-based emotion recognition has been adopted as a key tool to characterize human internal states across domains such as healthcare, education, and customer service [1,2]. Recent studies have demonstrated that facial expression recognition (FER) models can identify emotions with high accuracy; for instance, Safalı reported approximately 92% accuracy through a comparative analysis of different CNN architectures, highlighting the technical maturity of current FER systems [3].

Despite this momentum, current research faces fundamental constraints on further performance gains. Prior work has largely emphasized increasing deep learning model complexity, whereas improvements achievable through algorithmic refinement alone are approaching diminishing returns [4,5]. This has motivated a shift toward a “data-centric” paradigm that prioritizes the quality and consistency of training data over model architecture. Furthermore, affective data exhibit substantial inter-individual variability, and within an individual, the relationships among stimulus, subjective label, and facial expression are often inconsistent. Consequently, the key determinant of emotion recognition performance is no longer the sheer scale of data but the selective acquisition of high-quality data in which the internal affective state and external behavioral response are consistently aligned [6,7].

Facial expressions have long served as a core modality in emotion recognition research as representative nonverbal signals of internal affect. While early studies relied on small datasets in controlled settings, advances in large-scale databases and deep learning have established robust FER models as the dominant approach. Notably, Mollahosseini et al. constructed AffectNet by annotating more than one million web-collected images, providing a standard for in-the-wild facial expression computing [8]. However, many existing databases do not sufficiently control for subjective ambiguity during annotation and individual differences in expression. The fact that internal emotional states and facial expressions do not always coincide remains a major factor undermining the reliability of expression-based recognition [9].

To address these limitations, we propose emotional intelligence (EI) as a critical qualitative factor governing data consistency. EI is defined as an affective–cognitive competence that includes the ability to perceive, understand, and regulate emotional information [10,11,12]. This capacity is not merely a personality trait but a psychological information-processing capacity reflecting the efficiency of emotional regulation. Crucially, as shown by Lee et al., this regulation capacity is objectively reflected in autonomic nervous system activity, with heart rate variability (HRV) serving as a validated physiological marker of EI [13,14]. High-EI individuals, possessing superior emotional granularity, are more likely to perceive their internal states clearly and regulate their expressions appropriately, leading to a more consistent correspondence between subjective labels and facial patterns [15,16,17].

In this study, from a data-centric perspective, we analyze how EI levels influence the consistency of naturally elicited facial expression data and its subsequent impact on model performance. To ensure a controlled evaluation of data quality rather than architectural innovation, we employ an AffectNet-pretrained EfficientNetV2-L model as an objective “mechanical evaluator” to quantify the correspondence between estimated affective values and self-reported labels [15]. By utilizing a fixed, high-performance baseline model, we can isolate the effects of EI-based data characteristics on the accuracy and stability of emotion recognition. We propose the following hypotheses. First, a facial expression-based emotion recognition model trained on data from the High-EI group will achieve higher classification performance than a model trained on data from the Low-EI group. Second, even under conditions where the ambiguity of emotional expression increases, the model based on the High-EI group will exhibit higher performance and stability than the model based on the Low-EI group.

## 2. Methods

### 2.1. Participants

The target sample size was determined using G*Power 3.1 software based on an assumed effect size of dz = 0.6, a significance level of α ≤ 0.05, and a statistical power of 1 − β = 0.8 for a paired-sample analysis. Accordingly, 46 participants were initially recruited. All participants had normal or corrected-to-normal vision (≥0.6) and reported no history of neurological disorders in themselves or their first-degree family members. To minimize potential confounding effects on autonomic nervous system responses, participants were instructed to refrain from alcohol, caffeine, and nicotine intake from the day prior to the experiment and to obtain sufficient sleep.

Before participation, all individuals were informed of the general experimental procedure, excluding the specific purpose of the study to reduce expectancy bias, and provided written informed consent. All experimental procedures were approved by the Institutional Review Board of Sangmyung University (SMUIRB C-2021-001). Two participants were excluded due to technical issues during data acquisition and voluntary discontinuation. Consequently, data from 44 participants (18 males, 26 females; M = 23.6, SD = 4.8) were included in the final analysis.

This study was conducted as an exploratory pilot investigation under controlled laboratory conditions, during which synchronized electrocardiography (ECG) signals and high-definition facial videos were recorded.

### 2.2. Procedure

Each experimental session was conducted individually in an isolated, soundproof room to minimize external stimuli and potential distractions. Participants were seated comfortably at a distance of approximately 50 cm from a 27-inch LCD monitor (1920 × 1080 resolution, 60 Hz) and were instructed to minimize body movement to ensure the quality of the recorded neurophysiological and facial video data. For physiological monitoring, ECG sensors were attached using the standard limb lead I configuration, and cardiac activity was recorded continuously throughout the session.

**Figure 1:**
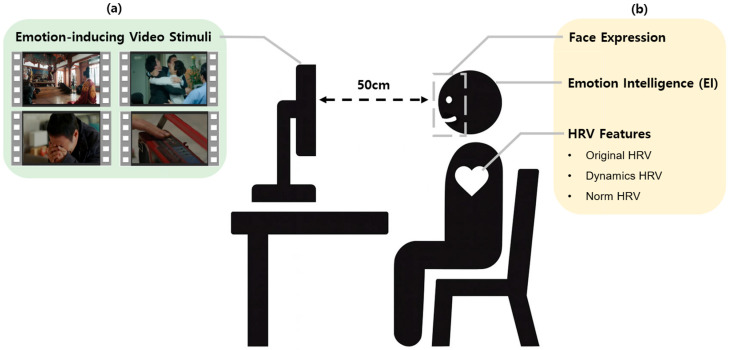
Experimental overview: (**a**) stimuli; (**b**) measured variables.

The experimental protocol consisted of two independent phases designed to separate participant grouping criteria from performance evaluation. The overall structure of the experimental protocol is illustrated in Figure 1, which summarizes (a) the stimulus sequence and (b) the measured variables across phases. In the first phase, participants’ EI levels were assessed using three validated procedures involving photographic and avatar-based stimuli, which targeted internal and external emotional awareness (see Section 2.2.1). The data obtained in this phase were used exclusively to categorize participants into High- and Low-EI groups.

Following a brief break, participants proceeded to the second phase, which comprised the emotion elicitation experiment. This phase aimed to collect naturally elicited facial expressions for subsequent model training and analysis. The session began with a 6 min neutral video to establish a physiological baseline, followed by four 6 min emotion-inducing video stimuli presented in a randomized order to mitigate sequence effects. To allow for emotional recovery and reduce carryover effects, a 3 min neutral video was inserted between consecutive stimuli. Detailed information regarding the elicitation stimuli is provided in Section 2.2.2.

During each 3 min neutral interval, participants performed a subjective affect evaluation of the preceding stimulus (see Section 2.2.3). Throughout the second phase, facial expressions were recorded in real time at 30 frames per second using a high-definition webcam mounted at the top center of the monitor, ensuring a frontal facial view suitable for accurate feature extraction.

#### 2.2.1. EI Assessment Procedure

Participants’ EI levels were assessed using the physiological evaluation framework proposed by Lee et al. [13]. This framework conceptualizes EI as a psychological information-processing capacity related to the perception of internal affective states and the interpretation of external emotional expressions. Objective physiological responses, specifically HRV, were employed as quantitative indicators to complement traditional questionnaire-based EI assessments.

The assessment consists of three task categories designed to evaluate self-awareness and others’ awareness using photographic and avatar-based stimuli: (1) self-awareness, (2) others’ awareness, and (3) emotion discrimination. In the self-awareness task, participants were presented with 40 images selected from the International Affective Picture System (IAPS). After each image, participants reported their momentary affective state according to four quadrants of the circumplex model: pleasant–arousal, pleasant–relaxation, unpleasant–arousal, and unpleasant–relaxation. This task was intended to assess the ability to associate internal affective experiences with multidimensional emotional representations.

The remaining two tasks evaluated the recognition and discrimination of external emotional expressions using avatar-based facial stimuli. These expressions were generated based on the Facial Action Coding System (FACS) and standardized across multiple intensity levels. In the others’ awareness task, participants identified the emotion category displayed by each avatar. In the emotion discrimination task, participants distinguished between subtle differences in emotion categories and intensity levels presented under varying conditions.

A standardized scoring scheme was applied across all three tasks. For each combination of evaluation index (3), emotion category (4), and stimulus instance (10), correct responses were assigned 10 points, yielding a total possible EI score ranging from 0 to 1200. Each evaluation index contributed a maximum of 400 points.

This assessment framework has been previously validated by comparing its score distribution with that of the Bar-On Emotional Quotient Inventory (EQ-i), a widely used self-report measure of EI. Prior validation results demonstrated that the HRV-based EI scores satisfied statistical normality assumptions and exhibited strong correlations with established psychological EI measures [13]. Based on this validated framework, participants in the present study were categorized according to their assessed EI levels.

#### 2.2.2. Emotion Elicitation Stimuli

For the emotion elicitation phase, a validated set of video stimuli developed by Lee et al. [13] was employed. The stimuli were designed to elicit spontaneous and natural facial expressions and consisted of video clips with a fixed duration of 6 min. Compared to brief or posed facial expression paradigms, longer video stimuli were used to allow participants sufficient time to enter a stable affective state and to capture temporally extended emotional responses.

**Figure 2:**
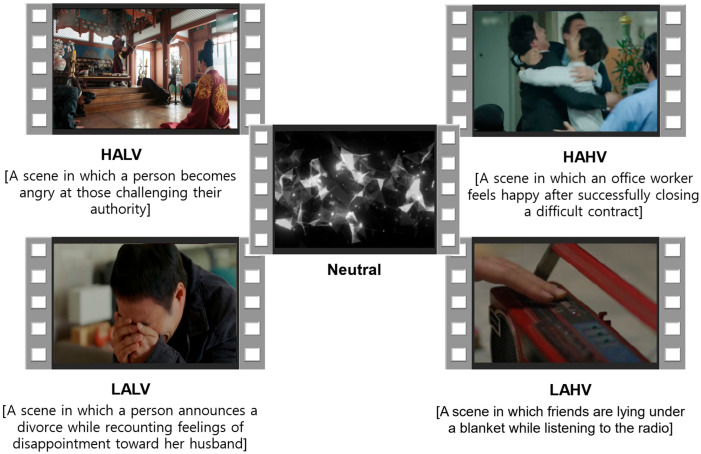
Images of the four emotion-elicitation stimulus videos and the neutral video.

The stimulus set comprised five videos for each of four emotion domains—High Arousal High Valence (HAHV), High Arousal Low Valence (HALV), Low Arousal High Valence (LAHV), and Low Arousal Low Valence (LALV)—resulting in a total of 20 stimulus videos. Representative images from the four emotion-elicitation stimulus videos, along with the neutral video, are illustrated in Figure 2. All videos were selected following the validation procedure described in the original study [13].

To support consistent facial expression elicitation, an additional selection criterion was applied based on facial expression prevalence. Specifically, only videos in which the target facial expressions were present in more than 50% of the frames, as verified in the original validation study, were included. This criterion was applied to reduce ambiguity in the emotional content of the stimuli and to ensure sustained presentation of emotion-relevant visual cues.

Each emotion-eliciting video was embedded within a standardized temporal structure consisting of a 6 min neutral baseline period, a 6 min emotion elicitation period, and a 3 min neutral recovery period. The neutral videos consisted of non-structured, achromatic visual patterns without identifiable objects or semantic content and were used to minimize affective stimulation while maintaining visual engagement. This structure allowed for the observation of baseline activity, stimulus-evoked responses, and post-stimulus recovery within a single experimental session.

Previous studies employing this stimulus set and experimental configuration reported significant differences in multiple HRV indices—including time-domain, frequency-domain, and dynamic variability measures—between High- and Low-EI groups [13,18]. Based on these prior validations, the same stimuli and temporal configuration were adopted in the present study.

#### 2.2.3. Subjective Affect Evaluation and Labeling

To assess subjective affective responses, participants rated their experienced levels of arousal and valence using a 7-point Likert scale immediately after each emotion elicitation stimulus. For the arousal dimension, a score of 1 indicated low arousal (relaxation), and a score of 7 indicated high arousal. For the valence dimension, a score of 1 represented low valence (unpleasant), and a score of 7 represented high valence (pleasant). A score of 4 on either dimension was defined as a neutral or indeterminate affective state.

Subjective ratings were used to generate response-driven labels rather than relying exclusively on the predefined emotional domains of the stimulus videos. This approach allowed the affective labels to reflect participants’ reported experiences. For binary classification, arousal ratings of 1–3 were categorized as low arousal (LA), and ratings of 5–7 as high arousal (HA). Similarly, valence ratings of 1–3 were categorized as low valence (LV), and ratings of 5–7 as high valence (HV). Because each stimulus video yielded ratings for both dimensions, the same video instances were included in both arousal-based and valence-based analyses.

Responses with a neutral rating of 4 were retained for analysis rather than excluded. While such responses have often been removed in prior emotion recognition studies [8], they were included in the present study to enable examination of data consistency across stimulus characteristics, subjective evaluation, and behavioral responses. To maintain a complete dataset for model training and comparison, neutral samples were assigned weak labels based on the predefined emotion domain of the corresponding stimulus video.

Using this labeling scheme, subjective evaluations were analyzed in conjunction with facial expression data to examine variations in response consistency across participants. Differences in the distribution of neutral responses and the alignment between stimulus properties, subjective ratings, and facial expressions were subsequently evaluated in relation to EI grouping.

## 3. Analysis

### 3.1. EI Group Classification

To examine differences in affective expression patterns across levels of EI, participants were divided into High-EI and Low-EI groups based on their EI assessment scores. Rather than treating EI as a psychometric ground truth, this grouping was employed as an operational stratification strategy to analyze variations in behavioral and physiological response consistency.

The cutoff score for group assignment was determined by jointly considering two criteria: (i) the stability of group-wise differences in involuntary physiological responses across emotional conditions and (ii) the maintenance of a reasonably balanced sample size between groups to preserve statistical power. This approach was motivated by prior findings that autonomic nervous system (ANS) responses, such as HRV, exhibit regulatory characteristics distinct from facial expressions, which are more susceptible to intentional modulation [9,18].

Facial expression signals were retained as the primary modality of interest in subsequent analyses, while HRV features were used as auxiliary indicators to assess whether candidate groupings exhibited systematic differences in physiological reactivity patterns. Importantly, HRV was not treated as a direct measure of EI but as an independent physiological channel to evaluate the consistency of group separation across emotional conditions.

EI cutoff values within the score range of 0–1200 were iteratively examined at fixed intervals. For each candidate cutoff, two quantitative measures were computed. First, a two-way analysis of variance (ANOVA) with Type III sums of squares and sum-to-zero contrasts was conducted for each HRV variable, with EI group (High vs. Low) and emotion label as factors. The number of HRV variables exhibiting a statistically significant interaction effect between the EI group and emotion label was recorded. Such interaction effects indicate that physiological responses to emotional stimuli differ in pattern across the two groups.

Second, group size imbalance was evaluated by calculating the difference in the number of participants assigned to each EI group. This measure was used to avoid extreme skewness that could compromise statistical power or model robustness in subsequent analyses.

Based on these criteria, the optimal cutoff was determined by identifying the threshold that maximized the number of HRV variables exhibiting significant interaction effects while preserving an acceptable balance in group sizes. The empirical results of this selection procedure are presented in the Results section.

### 3.2. Facial Expression Data

#### 3.2.1. Preprocessing and Data Extraction

**Figure 3:**
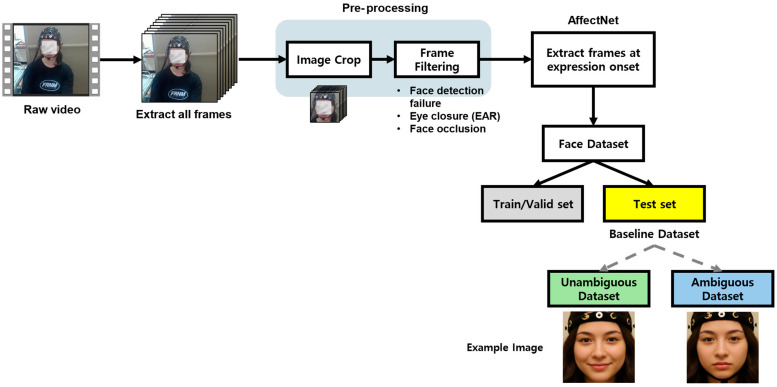
Facial expression data extraction pipeline. Gray boxes indicate the training and validation datasets; yellow boxes represent the baseline test dataset; green boxes denote the unambiguous test dataset; and blue boxes correspond to the ambiguous test dataset.

**Figure 4:**
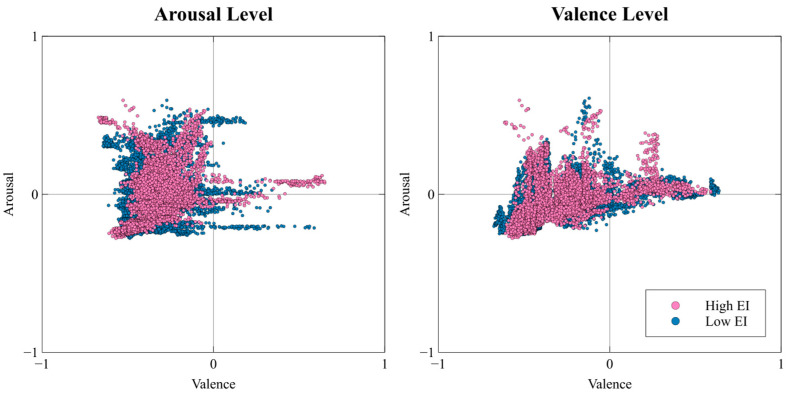
AffectNet-based arousal and valence coordinate distribution of the extracted facial expression dataset.

Figure 3 illustrates the overall facial expression data extraction procedure, and Figure 4 presents the arousal–valence coordinate distribution of the final selected facial frames. Facial expression data were collected by recording participants’ naturally occurring facial responses while they viewed the emotion-elicitation stimulus videos. Recordings were obtained using a webcam (Logitech HD 1080p, Lausanne, Switzerland).

For each participant, four face videos corresponding to the four emotion-elicitation conditions were recorded, each with a duration of 6 min. All videos were captured at a resolution of 640 × 480 pixels and a frame rate of 30 frames per second. Video-level emotion labels for arousal (low arousal vs. high arousal) and valence (low valence vs. high valence) were assigned based on the subjective evaluations described in Section 2.2.3.

Following frame extraction, an automatic preprocessing pipeline was applied to exclude frames that could compromise analysis reliability. Frames were removed if face detection failed, if facial feature estimation confidence was low (e.g., due to eye closure), or if the face was partially or fully occluded by hands or external objects.

For the remaining valid frames, continuous arousal and valence values in the range of −1 to 1 were estimated using an EfficientNetV2-L-based model pretrained on the AffectNet dataset [15]. In the present study, these estimates were treated as model-derived indicators reflecting the relative direction (sign) and magnitude of affective expressions inferred from facial appearance, rather than as direct measurements of participants’ internal emotional states.

To construct a facial expression dataset with a reduced proportion of incongruent frames, a two-stage selection procedure was applied. First, frames were retained if the sign of the estimated arousal or valence value matched the direction of the corresponding video-level subjective label. Second, among the remaining frames, those with larger absolute arousal or valence values were prioritized. Specifically, for each video, the top 100 frames ranked by arousal magnitude and the top 100 frames ranked by valence magnitude were selected, resulting in 200 frames per video. This procedure yielded a total of 800 facial frames per participant (4 videos × 200 frames).

The example images of the unambiguous and ambiguous datasets shown in Figure 3 are illustrative samples generated using generative artificial intelligence (Google Gemini 3 Flash). To create these illustrative images, representative facial images from the corresponding unambiguous/ambiguous datasets were provided to the GenAI tool as input, and the tool was instructed to generate new images that preserve the same facial expression patterns while replacing the identity of the face. These images were synthesized to reflect the statistical characteristics and distribution observed in the experimental facial expression data and were provided in place of the original facial images to protect participant privacy and portrait rights. The illustrative images were used solely for visualization purposes and were not included in the quantitative analysis or model training.

#### 3.2.2. Datasets

For model training and evaluation, a participant-specific facial expression dataset was constructed, as described in Section 3.2.1, yielding 800 facial frames per participant. This dataset was used for training and validating the single facial expression model described in Section 3.3 and Section 3.5.

To prevent data leakage, the full dataset was partitioned into training, validation, and test sets at the participant level, ensuring that facial frames from the same participant did not appear in more than one subset (Figure 3). This participant-wise separation was applied throughout all experiments.

The test set was constructed by selecting four participants in total, including two from the High-EI group and two from the Low-EI group, to reduce potential bias associated with the EI group composition. This test set was fixed across all experiments and used as a common reference for performance comparison under identical evaluation conditions, regardless of differences in training data composition or EI group.

The remaining participants, excluding those assigned to the test set, were divided into training and validation sets. A stratified group split with an 8:2 ratio was applied while preserving participant boundaries, such that the distribution of binary affective labels remained comparable between the two sets. This procedure was used to reduce overfitting to participant-specific facial characteristics or recording conditions and to support generalization based on affective features reflected in facial expressions.

In addition, two evaluation subsets were derived from the fixed test set to examine performance differences associated with data consistency and ambiguity. In this study, consistency and ambiguity were operationally defined based on the relationship between (i) the direction of the subjective label and the direction (sign) of the arousal or valence value estimated by the pretrained facial expression model and (ii) the magnitude (absolute value) of the estimated score.

The unambiguous subset consisted of facial frames for which the estimated sign matched the direction of the corresponding subjective label and exhibited larger absolute estimated values. The ambiguous subset consisted of frames that either showed a mismatch between the estimated sign and the subjective label direction or exhibited smaller absolute estimated values.

To ensure comparability between subsets, the same sampling unit was applied. Specifically, six facial frames were selected from each video for each participant, resulting in 24 frames per participant across the four videos. Because the test set included four participants, each subset (unambiguous and ambiguous) contained a total of 96 frames (4 participants × 4 videos × 6 frames). This design enabled comparison across consistency conditions while controlling for differences in sample size.

### 3.3. Model Architecture

The facial expression model used in this study was designed to perform two learning objectives in parallel: a binary classification task and a regression task, both operating on the same affective dimension. Given an input facial image, the classification task predicts either arousal versus relaxation (arousal model) or pleasant versus unpleasant (valence model), while the regression task estimates a continuous value corresponding to the same dimension. This joint formulation was adopted to incorporate continuous affective information into representation learning while maintaining a discrete decision output for evaluation.

As the backbone network, EfficientNetV2-L was employed. This architecture has been reported to exhibit stable performance across multiple facial expression recognition benchmarks and was used in the present study as a fixed feature extraction backbone rather than as a target of architectural optimization. The backbone produces a high-dimensional feature representation from the input image, which is shared by both the classification and regression heads.

From a single input image, the model generates two outputs in parallel. The binary classification head consists of a single fully connected layer that projects the backbone feature vector to a one-dimensional logit, followed by a sigmoid function to estimate the probability of the target class (arousal or pleasant). The regression head consists of a separate fully connected layer that maps the same feature vector to a continuous arousal or valence value. The regression output was used during training to provide an auxiliary learning signal and was not directly used for final classification.

Training labels were assigned at the frame level using the subjective evaluation-based labeling procedure described in Section 2.2.3. For the arousal model, low arousal (LA) and high arousal (HA) were encoded as 0 and 1, respectively, and for the valence model, low valence (LV) and high valence (HV) were encoded as 0 and 1. For the regression task, target values were set to the continuous arousal or valence estimates obtained from the pretrained AffectNet-based model. These continuous values were treated as model-derived reference signals to guide representation learning, rather than as direct measurements of participants’ internal affective states.

All facial images were resized to 224 × 224 pixels and normalized using channel-wise means ([0.485, 0.456, 0.406]) and standard deviations ([0.229, 0.224, 0.225]). During training, data augmentation was applied with a fixed probability, including random grayscale conversion, brightness jitter (range: 0.8–1.2), and Gaussian blur (intensity: 0.1–1.5). No augmentation was applied to validation or test images; only resizing and normalization were performed to maintain distributional consistency during evaluation.

**Figure 5:**
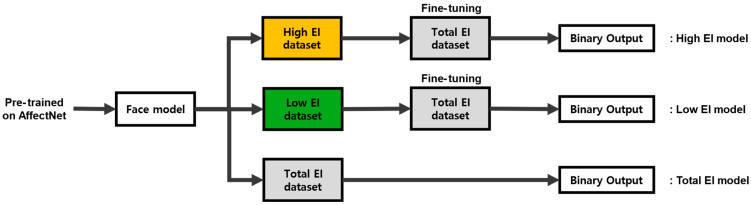
Model training pipeline by EI level. Color coding indicates dataset composition: yellow boxes correspond to the High-EI dataset, green boxes to the Low-EI dataset, and gray boxes to the combined High- and Low-EI dataset.

To examine the influence of EI-related data composition on model behavior, separate training datasets were constructed for the High-EI, Low-EI, and Total-EI groups. Independent models were first trained on each dataset using AffectNet-pretrained weights as initialization (Figure 5). The resulting weights were saved as EI-specific pretrained models. Subsequently, models initialized from the High-EI and Low-EI pretrained weights were further trained on the Total-EI dataset, while a reference model was trained on the Total-EI dataset alone without EI-based initialization. This procedure enabled comparison across different initialization conditions while holding the model architecture constant.

**Figure 6:**
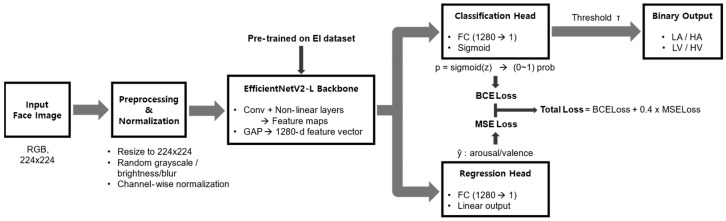
Single facial expression model architecture.

Model training followed a two-stage fine-tuning strategy (Figure 6). In Stage 1, the EfficientNetV2-L backbone was frozen, and only the classification and regression heads were trained for 4 epochs with a learning rate of 3 × 10^−4^. In Stage 2, the full network was fine-tuned for an additional 6 epochs. The backbone learning rate was initialized at 2 × 10^−5^ and increased to a base rate of 1 × 10^−4^, while the heads retained a learning rate of 3 × 10^−4^. Optimization was performed using AdamW with a weight decay coefficient of 1 × 10^−2^ and a batch size of 32. Gradient clipping with an ℓ_2_-norm threshold of 1.0 was applied throughout training. A learning rate schedule combining linear warm-up and cosine annealing was used in both stages, with linear warm-up applied over the first three epochs of each stage.

The overall loss function was defined as a weighted sum of the binary classification loss and the regression loss. Binary classification was optimized using BCEWithLogitsLoss function implemented in PyTorch (version 2.6.0, CUDA 11.8), with class weighting applied to address label imbalance., Regression was optimized using mean squared error (MSE). The total loss was defined as

(1) $$L=L_{BCE}+\lambda L_{MSE}$$

where the regression weight λ was set to 0.4.

For the final class assignment, the sigmoid output of the classification head was converted to binary labels using a decision threshold selected on the validation set. Candidate thresholds were uniformly sampled from the range 0.2 to 0.8, and the threshold that maximized the macro F1-score was selected. This threshold was then applied consistently across the training, validation, and test sets.

### 3.4. Statistical Analysis

Group differences in model performance were evaluated in terms of discrimination and classification correctness. All analyses were conducted separately for each evaluation condition (baseline, unambiguous, and ambiguous) and recognition task (arousal and valence). For each training group, five independent training repetitions were performed while maintaining an identical fixed test set across repetitions.

Discrimination performance was quantified using the area under the receiver operating characteristic curve (ROC-AUC). Within each training repetition, ROC-AUC values of the High-EI model were compared with those of the Low-EI and Total-EI models using paired DeLong tests, because competing models were evaluated on the same test items and therefore produced statistically dependent AUC estimates. For each repetition, the difference in AUC (ΔAUC), its standard error, and the corresponding 95% confidence interval were derived from the DeLong covariance estimate. To summarize evidence across repetitions, *p*-values were combined using Fisher’s method, treating repetition-level results as independent, given independently trained models. In addition, ΔAUC estimates were pooled using a fixed-effect inverse-variance approach based on repetition-specific standard errors to obtain an overall ΔAUC with a 95% confidence interval.

Classification correctness was analyzed at the test-item level as a binary outcome (correct vs. incorrect). Because identical test items were evaluated repeatedly across the five training repetitions, a binomial generalized estimating equation (GEE) model was fitted to account for within-item correlation. The training group was included as a categorical predictor with High EI as the reference category, and training repetition was included as a fixed effect. An exchangeable working correlation structure was specified with clustering at the test-item level. Results are reported as odds ratios (ORs) with 95% confidence intervals based on robust standard errors.

To control for multiple testing, the Benjamini–Hochberg false discovery rate (FDR) procedure was applied within each family of statistical comparisons.

### 3.5. External Transfer-Learning-Based Model Training

To assess the robustness of the findings across modeling frameworks, an additional set of models was trained using an independent transfer-learning pipeline based on a publicly available facial expression recognition framework [19,20]. The same dataset, labels, and evaluation conditions (baseline, unambiguous, and ambiguous) were used to ensure direct comparability with the primary analysis.

All images were resized to a fixed resolution and normalized using ImageNet statistics. Training data were augmented using standard photometric transformations. For each recognition task (arousal and valence), data were partitioned using stratified group-wise five-fold cross-validation, with subject identity treated as the grouping variable, preventing subject overlap between training and validation subsets within each fold.

Models were initialized with ImageNet-pretrained convolutional backbones, and task-specific prediction layers were trained using a staged fine-tuning procedure in which newly initialized layers were optimized prior to the gradual unfreezing of backbone layers. Training employed a binary classification objective with an auxiliary regression term based on continuous annotations, and class imbalance was handled through fold-specific loss weighting derived from the training data.

Within each fold, model selection was performed using the validation macro F1-score, and the classification threshold was determined from validation predictions. The selected model from each fold was subsequently evaluated on the held-out test set to obtain fold-wise test predictions. Test-set predictions obtained from the five folds were aggregated and analyzed using the same statistical procedures described in Section 3.4.

## 4. Results

### 4.1. Determination of the Optimal EI Cutoff

The iterative evaluation of candidate EI cutoff scores revealed that a threshold of 830 provided the most appropriate balance between physiological differentiation and group size stability. At this cutoff, the number of HRV variables exhibiting significant interaction effects between the EI group and emotion label was relatively high compared to neighboring thresholds, indicating distinct patterns of physiological reactivity across emotional conditions.

**Figure 7:**
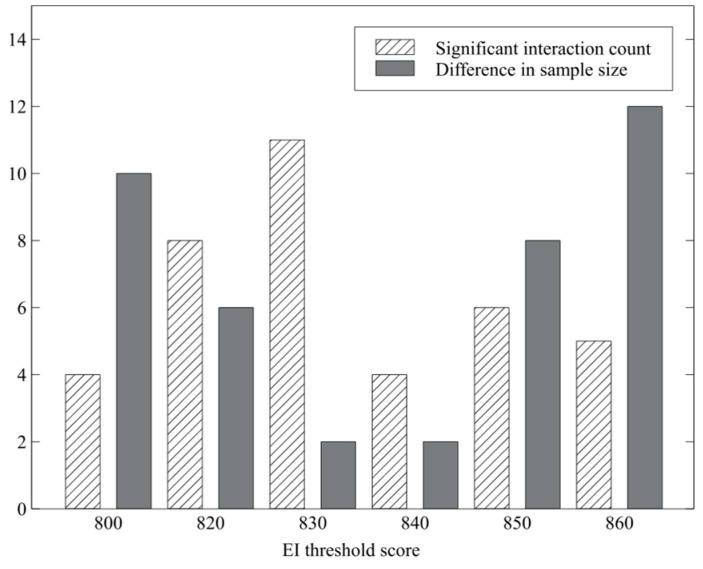
Criteria for EI group classification.

Importantly, this threshold also maintained a reasonably balanced distribution of participants between the High-EI and Low-EI groups, thereby reducing potential bias due to sample size imbalance and preserving statistical power for subsequent analyses (Figure 7).

Accordingly, participants with EI scores above 830 were assigned to the High-EI group, whereas those with scores of 830 or below were assigned to the Low-EI group. Descriptive statistics for each group, including sample sizes and mean subjective evaluation scores, are summarized in Table 1.

### 4.2. Recognition Performance Across Dataset Conditions

The performance of the single facial expression model across three evaluation conditions (baseline, unambiguous, and ambiguous) and two binary recognition tasks (arousal: LA–HA; valence: LV–HV) is summarized in Table 2 and Table 3. Table 2 presents macro-averaged precision, recall, F1-score, and accuracy, and Table 3 reports ROC-AUC and PR-AUC values. All metrics are reported as mean (SD) across five independent training repetitions evaluated on an identical, fixed test set. The model architecture and training procedure were held constant across experiments, and only the composition of the training data (High EI, Low EI, or Total EI) differed.

On the baseline dataset, for arousal recognition, the High-EI model achieved a macro F1-score of 0.9105 ± 0.0237, compared with 0.8914 ± 0.0291 (Low EI) and 0.8931 ± 0.0397 (Total EI). The corresponding accuracy values were 0.9118 ± 0.0229 (High EI), 0.8940 ± 0.0263 (Low EI), and 0.8954 ± 0.0366 (Total EI). The ROC-AUC values were 0.9787 ± 0.0108 (High EI), 0.9719 ± 0.0108 (Low EI), and 0.9702 ± 0.0200 (Total EI), and the PR-AUC values were 0.9843 ± 0.0076, 0.9788 ± 0.0082, and 0.9783 ± 0.0143, respectively. For valence recognition in the baseline condition, macro F1-scores were 0.9358 ± 0.0078 (High EI), 0.9283 ± 0.0300 (Low EI), and 0.9367 ± 0.0322 (Total EI), with accuracy values of 0.9361 ± 0.0078, 0.9286 ± 0.0302, and 0.9369 ± 0.0323, respectively. The ROC-AUC values were 0.9963 ± 0.0037 (High EI), 0.9958 ± 0.0044 (Low EI), and 0.9969 ± 0.0056 (Total EI), and the PR-AUC values were 0.9954 ± 0.0045, 0.9948 ± 0.0054, and 0.9963 ± 0.0065, respectively.

On the Unambiguous dataset, for arousal recognition, macro F1-scores were 0.9518 ± 0.0203 (High EI), 0.9539 ± 0.0174 (Low EI), and 0.9318 ± 0.0435 (Total EI), with corresponding accuracy values of 0.9521 ± 0.0203, 0.9542 ± 0.0174, and 0.9333 ± 0.0407. The ROC-AUC values were 0.9989 ± 0.0019, 0.9983 ± 0.0019, and 0.9968 ± 0.0064, and the PR-AUC values were 0.9992 ± 0.0013, 0.9988 ± 0.0014, and 0.9978 ± 0.0043, respectively. For valence recognition in the unambiguous condition, macro F1-scores were 0.9518 ± 0.0203 (High EI), 0.9539 ± 0.0281 (Low EI), and 0.9540 ± 0.0300 (Total EI), with accuracy values of 0.9521 ± 0.0203, 0.9542 ± 0.0281, and 0.9542 ± 0.0300, respectively. The ROC-AUC values were 0.9992 ± 0.0007 (High EI), 0.9994 ± 0.0006 (Low EI), and 0.9993 ± 0.0013 (Total EI), and the PR-AUC values were 0.9990 ± 0.0008, 0.9992 ± 0.0007, and 0.9992 ± 0.0015, respectively.

On the ambiguous dataset, for arousal recognition, macro F1-scores were 0.8745 ± 0.0379 (High EI), 0.8562 ± 0.0521 (Low EI), and 0.8621 ± 0.0444 (Total EI), with corresponding accuracy values of 0.8771 ± 0.0349, 0.8604 ± 0.0469, and 0.8646 ± 0.0417, respectively. The ROC-AUC values were 0.9621 ± 0.0199 (High EI), 0.9525 ± 0.0193 (Low EI), and 0.9480 ± 0.0331 (Total EI), and the PR-AUC values were 0.9725 ± 0.0151, 0.9650 ± 0.0143, and 0.9637 ± 0.0224, respectively. For valence recognition in the ambiguous condition, macro F1-scores were 0.9184 ± 0.0137 (High EI), 0.9101 ± 0.0379 (Low EI), and 0.9186 ± 0.0431 (Total EI), with accuracy values of 0.9188 ± 0.0136, 0.9104 ± 0.0380, and 0.9188 ± 0.0432, respectively. The ROC-AUC values were 0.9913 ± 0.0070 (High EI), 0.9906 ± 0.0086 (Low EI), and 0.9925 ± 0.0094 (Total EI), and the PR-AUC values were 0.9896 ± 0.0082, 0.9890 ± 0.0100, and 0.9915 ± 0.0102, respectively.

Across evaluation conditions, performance was highest in the unambiguous dataset, followed by the baseline and ambiguous datasets.

### 4.3. Statistical Analysis Results

Group differences were evaluated using paired DeLong tests for ROC-AUC and binomial generalized estimating equation (GEE) models accounting for clustering at the test-item level.

In the baseline condition, mean ROC-AUC values for arousal classification were 0.9787 ± 0.0108 (High EI), 0.9719 ± 0.0108 (Low EI), and 0.9702 ± 0.0200 (Total EI). Meta-analytic aggregation of repetition-level DeLong tests showed statistically significant differences after FDR correction for both High vs. Low (pooled ΔAUC = 0.0005, 95% CI [−0.0010, 0.0020], q < 0.001) and High vs. Total (pooled ΔAUC = −0.0015, 95% CI [−0.0025, −0.0005], q < 0.001).

For baseline valence classification, mean ROC-AUC values were 0.9963 ± 0.0037 (High EI), 0.9958 ± 0.0044 (Low EI), and 0.9969 ± 0.0056 (Total EI). The High vs. Low comparison was statistically significant after FDR correction (pooled ΔAUC = −0.0012, 95% CI [−0.0014, −0.0009], q < 0.001), whereas the High vs. Total comparison was not statistically significant after FDR correction.

GEE analyses in the baseline condition showed significant group effects. For arousal classification, Low-EI-trained models had lower odds of correct classification relative to High-EI-trained models (OR = 0.8151, 95% CI [0.7669, 0.8663], q < 0.001), and Total-EI-trained models also showed reduced odds (OR = 0.8271, 95% CI [0.7818, 0.8751], q < 0.001). For valence classification, Low-EI-trained models had lower odds of correct classification compared with High-EI-trained models (OR = 0.8872, 95% CI [0.8292, 0.9493], q = 0.002), whereas the Total-EI comparison was not statistically significant after FDR correction.

In the ambiguous condition, Fisher-combined DeLong tests indicated statistically significant differences for arousal classification in both High vs. Low and High vs. Total comparisons (q < 0.001). The corresponding inverse-variance pooled ΔAUC estimates had 95% confidence intervals that included zero. No statistically significant group differences were observed in GEE analyses for this condition after FDR correction.

In the unambiguous condition, no statistically significant group differences were detected in either ROC-AUC comparisons or GEE analyses after FDR correction. Table 4 summarizes the statistical comparison results for discrimination and classification correctness across training groups.

### 4.4. Results with Alternative Backbone Models

Additional backbone networks (ResNet50 and VGG16) were trained and evaluated on the baseline dataset under the same experimental and statistical procedures described in Section 3.4. Performance metrics are summarized in Table 5, and statistical comparisons are reported in Table 6. All values are reported as mean (SD) across five independent training repetitions, evaluated on an identical fixed test set.

For the ResNet50 backbone in the arousal task, the High-EI model achieved a macro F1-score of 0.4577 ± 0.0787 and an accuracy of 0.4715 ± 0.0826, compared with 0.4918 ± 0.0323 and 0.5094 ± 0.0386 for the Low-EI model and 0.4723 ± 0.0200 and 0.4910 ± 0.0319 for the Total-EI model. The corresponding ROC-AUC values were 0.4686 ± 0.0999 (High EI), 0.5199 ± 0.0351 (Low EI), and 0.4868 ± 0.0395 (Total EI), and the PR-AUC values were 0.5694 ± 0.0481, 0.5991 ± 0.0335, and 0.5787 ± 0.0372, respectively. In the valence task, macro F1-scores were 0.6812 ± 0.0508 (High EI) and 0.6595 ± 0.0389 (Low EI), with corresponding accuracy values of 0.6936 ± 0.0385 and 0.6721 ± 0.0325, respectively. ROC-AUC values were 0.7635 ± 0.0288 (High EI) and 0.7402 ± 0.0350 (Low EI), and PR-AUC values were 0.6585 ± 0.0428 and 0.6449 ± 0.0331 (Table 5).

Paired DeLong tests with repetition-level aggregation identified statistically significant differences between training groups (Table 6). For ResNet50 in the arousal task, the High vs. Low comparison yielded a pooled ΔAUC of −0.0107 (95% CI −0.0170 to −0.0045, q < 0.001), and the High vs. Total comparison yielded a pooled ΔAUC of 0.0067 (95% CI 0.0018 to 0.0116, q < 0.001). In the valence task, the High vs. Low comparison was statistically significant (pooled ΔAUC = 0.0043, 95% CI −0.0003 to 0.0088, q < 0.001), as was the High vs. Total comparison (pooled ΔAUC = 0.0141, 95% CI 0.0104 to 0.0178, q < 0.001). For VGG16 in the arousal task, the High vs. Low comparison was statistically significant (pooled ΔAUC = 0.0059, 95% CI 0.0021 to 0.0097, q < 0.001).

GEE analyses of classification correctness showed significant group effects (Table 6). For ResNet50 in the arousal task, the odds of correct classification differed between High EI and Low EI (OR = 1.1642, 95% CI 1.1155 to 1.2149, q < 0.001) and between High EI and Total EI (OR = 1.0815, 95% CI 1.0452 to 1.1190, q < 0.001). In the valence task, the High vs. Low comparison was statistically significant (OR = 0.9053, 95% CI 0.8663 to 0.9460, q < 0.001), whereas the High vs. Total comparison was not statistically significant after FDR correction (q = 0.290). For VGG16 in the arousal task, the High vs. Low comparison was statistically significant (OR = 0.8915, 95% CI 0.8593 to 0.9248, q < 0.001).

## 5. Discussion

This study examined whether the composition of training data, stratified by emotional intelligence (EI), systematically influences facial expression recognition under naturally elicited conditions, while holding model architecture and training procedures constant. Across evaluation contexts, EI-stratified training was associated with measurable differences in model performance, particularly under baseline and, to a lesser extent, ambiguous conditions.

Descriptive results indicated that, in the Baseline dataset, models trained on High-EI-derived data showed higher performance in arousal recognition relative to models trained on Low EI or Total EI data. In contrast, performance differences were minimal under unambiguous conditions, where all models approached ceiling levels. Under ambiguous conditions, overall performance declined, and numerical differences favoring High-EI-trained models were observed in arousal recognition.

The formal statistical aggregation across independent training repetitions refines this pattern. In the baseline condition, paired DeLong meta-analyses identified statistically significant differences in discrimination (ROC-AUC) between training groups after FDR correction, although pooled ΔAUC estimates were small in magnitude. In parallel, item-level GEE models revealed significant differences in classification correctness, particularly for arousal recognition, indicating that training data composition influenced the probability of correct classification at the test-item level. In the ambiguous condition, some ROC-AUC comparisons reached statistical significance when repetition-level evidence was combined, whereas pooled confidence intervals for ΔAUC included zero, and GEE analyses did not detect reliable differences. No statistically significant group differences were observed under unambiguous conditions. Together, these findings indicate that EI-related effects are most detectable when stimuli provide moderate affective signal strength and diminish when signal clarity is high.

Importantly, these effects were not confined to a single network architecture. When the same EI-stratified protocol was applied to alternative backbones (ResNet50 and VGG16) [19,20], statistically significant differences between training groups were again observed in several task–architecture combinations. Although effect sizes remained modest, the presence of EI-related differences across distinct convolutional backbones suggests that the phenomenon is not an artifact of a specific model configuration.

From a theoretical perspective, these findings are consistent with the literature linking higher EI to enhanced sensitivity to affective cues, improved discrimination of complex or mixed expressions, and more adaptive emotion regulation [21,22]. Individuals with higher EI have been shown to detect emotional signals more rapidly and to allocate attention more efficiently to affectively salient facial regions [21]. Eye-tracking evidence further demonstrates systematic differences in gaze behavior across EI profiles, with machine learning models achieving high accuracy in predicting EI based on visual attention patterns [23]. Such differences may contribute to greater internal consistency between stimulus context, subjective evaluation, and expressed facial behavior in high-EI participants, potentially influencing the statistical structure of training data derived from these groups.

The present findings also extend our previous report [15], in which EI-related performance differences were observed across multiple deep learning architectures in the valence–arousal space. In the current study, repetition-level aggregation and item-level modeling provide a more granular characterization of these differences, showing that statistically detectable effects can coexist with small pooled discrimination differences. This distinction underscores the importance of examining both discrimination metrics and classification correctness when evaluating data-composition effects.

Data ambiguity represents a central constraint in naturalistic facial expression recognition. Real-world expressions frequently involve mixed affective states and variable intensities, increasing label uncertainty and reducing categorical separability. Recent work advocates probabilistic or soft-label approaches for representing such ambiguity [24]. Within this broader context, the observed modulation of EI-related effects by evaluation condition suggests that training data composition interacts with stimulus clarity. The wider FER literature further highlights challenges related to distributional shifts, contextual variability, and limited ecological validity of laboratory datasets [25,26]. These factors collectively emphasize that improvements in model robustness may depend not only on architectural refinement but also on the internal coherence and representational properties of the training data.

Overall, the results indicate that EI-stratified training data are associated with statistically detectable differences in both discrimination and classification correctness under specific evaluation contexts. Although the magnitude of pooled discrimination effects was generally small, the consistency of findings across independent repetitions and across alternative backbones supports the conclusion that training data composition constitutes a systematic factor in naturally elicited facial expression recognition. These findings align with data-centric perspectives in machine learning, highlighting that the statistical structure and internal consistency of training data can meaningfully shape model behavior alongside architectural design.

## 6. Conclusions

This study examined whether differences in data consistency associated with EI-based participant grouping translate into measurable changes in facial-expression-based emotion recognition under naturally elicited conditions within a data-centric framework. Identical binary classifiers for arousal (LA–HA) and valence (LV–HV) recognition were trained while varying only the composition of the training data (High EI, Low EI, or Total EI) and evaluated across baseline, unambiguous, and ambiguous conditions. EI-stratified training was associated with statistically detectable, context-dependent differences in performance: group differences were observed primarily under baseline conditions and, to a more limited extent, under ambiguous conditions, whereas no reliable differences were detected under unambiguous conditions. Although pooled discrimination differences (ROC-AUC) were generally small in magnitude, item-level analyses identified significant differences in classification correctness in specific task–condition combinations, and comparable patterns were observed across alternative backbone architectures, indicating that the effects were not confined to a single model implementation. These findings support the view that facial expression recognition performance in naturalistic settings is shaped not only by architectural design or dataset size but also by the statistical structure and internal coherence of the training data, particularly the alignment between subjective labels and expressed facial behavior. The conclusions are constrained by the laboratory-based elicitation context, the limited sample size, and the focus on convolutional backbone models; future research should validate the proposed EI-informed data-centric strategy in larger and more diverse populations, evaluate additional state-of-the-art recognition architectures and affect representation frameworks, and extend the analysis to multimodal signals to assess generalizability and reproducibility.

## Figures and Tables

**Table 1:** Sample size, mean EI score, and mean subjective evaluation scores by EI group (mean (SD)).

EI Group	*N*	EI Score	Subjective Score			
			LA	HA	LV	HV
High EI	23	930.00 (73.61)	2.45 (0.95)	5.48 (0.86)	2.83 (0.84)	6.16 (0.85)
Low EI	21	710.48 (124.68)	2.81 (0.98)	5.43 (0.80)	2.83 (0.76)	5.79 (0.87)
Total EI	44	825.23 (149.35)	2.61 (0.97)	5.46 (0.83)	2.83 (0.80)	5.98 (0.88)

**Table 2:** Emotion recognition performance of the single facial expression model across evaluation conditions (baseline, unambiguous, and ambiguous). Values are reported as mean (SD) across five independent training repetitions on an identical fixed test set for arousal and valence tasks (Prec.: macro precision; Rec.: macro recall; F1: macro F1-score; Acc.: accuracy).

EI Group		Baseline		Unambiguous		Ambiguous	
		Arousal	Valence	Arousal	Valence	Arousal	Valence
High EI	Prec.	0.9124 (0.0207)	0.9358 (0.0072)	0.9513 (0.0195)	0.9516 (0.0195)	0.8787 (0.0286)	0.9192 (0.0135)
	Rec.	0.9130 (0.0258)	0.9421 (0.0076)	0.9574 (0.0180)	0.9569 (0.0172)	0.8759 (0.0408)	0.9251 (0.0139)
	F1	0.9105 (0.0237)	0.9358 (0.0078)	0.9518 (0.0203)	0.9518 (0.0203)	0.8745 (0.0379)	0.9184 (0.0137)
	Acc.	0.9118 (0.0229)	0.9361 (0.0078)	0.9521 (0.0203)	0.9521 (0.0203)	0.8771 (0.0349)	0.9188 (0.0136)
Low EI	Prec.	0.8971 (0.0191)	0.9305 (0.0250)	0.9531 (0.0165)	0.9543 (0.0272)	0.8634 (0.0401)	0.9138 (0.0314)
	Rec.	0.8913 (0.0337)	0.9353 (0.0264)	0.9593 (0.0155)	0.9587 (0.0242)	0.8563 (0.0556)	0.9177 (0.0341)
	F1	0.8914 (0.0291)	0.9283 (0.0300)	0.9539 (0.0174)	0.9539 (0.0281)	0.8562 (0.0521)	0.9101 (0.0379)
	Acc.	0.8940 (0.0263)	0.9286 (0.0302)	0.9542 (0.0174)	0.9542 (0.0281)	0.8604 (0.0469)	0.9104 (0.0380)
Total EI	Prec.	0.8972 (0.0308)	0.9380 (0.0285)	0.9361 (0.0326)	0.9540 (0.0276)	0.8667 (0.0412)	0.9213 (0.0387)
	Rec.	0.8946 (0.0438)	0.9437 (0.0290)	0.9349 (0.0487)	0.9593 (0.0267)	0.8648 (0.0478)	0.9267 (0.0404)
	F1	0.8931 (0.0397)	0.9367 (0.0322)	0.9318 (0.0435)	0.9540 (0.0300)	0.8621 (0.0444)	0.9186 (0.0431)
	Acc.	0.8954 (0.0366)	0.9369 (0.0323)	0.9333 (0.0407)	0.9542 (0.0300)	0.8646 (0.0417)	0.9188 (0.0432)

**Table 3:** Discrimination performance of the single facial expression model (ROC-AUC and PR-AUC) across evaluation conditions. Values are reported as mean (SD) across five independent training repetitions on an identical fixed test set for arousal and valence tasks.

EI Group		Baseline		Unambiguous		Ambiguous	
		Arousal	Valence	Arousal	Valence	Arousal	Valence
High EI	ROC-AUC	0.9787 (0.0108)	0.9963 (0.0037)	0.9989 (0.0019)	0.9992 (0.0007)	0.9621 (0.0199)	0.9913 (0.0070)
	PR-AUC	0.9843 (0.0076)	0.9954 (0.0045)	0.9992 (0.0013)	0.9990 (0.0008)	0.9725 (0.0151)	0.9896 (0.0082)
Low EI	ROC-AUC	0.9719 (0.0108)	0.9958 (0.0044)	0.9983 (0.0019)	0.9994 (0.0006)	0.9525 (0.0193)	0.9906 (0.0086)
	PR-AUC	0.9788 (0.0082)	0.9948 (0.0054)	0.9988 (0.0014)	0.9992 (0.0007)	0.9650 (0.0143)	0.9890 (0.0100)
Total EI	ROC-AUC	0.9702 (0.0200)	0.9969 (0.0056)	0.9968 (0.0064)	0.9993 (0.0013)	0.9480 (0.0331)	0.9925 (0.0094)
	PR-AUC	0.9783 (0.0143)	0.9963 (0.0065)	0.9978 (0.0043)	0.9992 (0.0015)	0.9637 (0.0224)	0.9915 (0.0102)

**Table 4:** Statistical comparisons of discrimination (ROC–AUC) and classification correctness across training groups using paired DeLong tests with repetition-level aggregation and binomial generalized estimating equations (GEEs).

Test Dataset		High EI vs.	DeLong Test			Binomial GEE		
			Pooled ΔAUC	95% CI	q (FDR)	Odds Ratio	95% CI	q (FDR)
Baseline	arousal	Low EI	0.0005	[−0.0010, 0.0020]	<0.001	0.8151	[0.7669, 0.8663]	<0.001
		Total EI	−0.0015	[−0.0025, −0.0005]	<0.001	0.8271	[0.7818, 0.8751]	<0.001
	valence	Low EI	−0.0012	[−0.0014, −0.0009]	<0.001	0.8872	[0.8292, 0.9493]	0.002
		Total EI	−0.0002	[−0.0004, 0.0000]	0.018	1.0128	[0.9513, 1.0781]	0.879
Ambiguous	arousal	Low EI	0.0005	[−0.0090, 0.0099]	<0.001	0.8627	[0.7181, 1.0364]	0.275
		Total EI	−0.0007	[−0.0064, 0.0050]	<0.001	0.8938	[0.7341, 1.0883]	0.528
	valence	Low EI	−0.0019	[−0.0036, −0.0002]	0.052	0.8983	[0.7176, 1.1254]	0.599
		Total EI	0.0004	[−0.0011, 0.0020]	0.052	1	[0.8023, 1.2464]	1
Unambiguous	arousal	Low EI	−0.0002	[−0.0014, 0.0009]	0.363	1.0483	[0.8928, 1.2310]	0.847
		Total EI	−0.0005	[−0.0013, 0.0004]	0.293	0.7018	[0.4773, 1.0319]	0.216
	valence	Low EI	−0.0003	[−0.0011, 0.0004]	0.594	1.0479	[0.6970, 1.5756]	0.897
		Total EI	−0.0005	[−0.0015, 0.0004]	0.44	1.0479	[0.8012, 1.3706]	0.879

**Table 5:** Performance of alternative backbone models (ResNet50, VGG16) on the baseline dataset. Values are reported as mean (SD) across five independent training repetitions on an identical fixed test set (F1: macro F1-score; Acc.: accuracy).

EI Group		ResNet50		VGG16	
		Arousal	Valence	Arousal	Valence
High EI	F1	0.4577 (0.0787)	0.6812 (0.0508)	0.5389 (0.0247)	0.8040 (0.0245)
	Acc.	0.4715 (0.0826)	0.6936 (0.0385)	0.5505 (0.0278)	0.8066 (0.0257)
	ROC-AUC	0.4686 (0.0999)	0.7635 (0.0288)	0.5655 (0.0424)	0.9019 (0.0161)
	PR-AUC	0.5694 (0.0481)	0.6585 (0.0428)	0.6120 (0.0249)	0.8851 (0.0151)
Low EI	F1	0.4918 (0.0323)	0.6595 (0.0389)	0.5173 (0.0322)	0.7743 (0.0353)
	Acc.	0.5094 (0.0386)	0.6721 (0.0325)	0.5220 (0.0349)	0.7785 (0.0326)
	ROC-AUC	0.5199 (0.0351)	0.7402 (0.0350)	0.5715 (0.0253)	0.8824 (0.0430)
	PR-AUC	0.5991 (0.0335)	0.6449 (0.0331)	0.6160 (0.0292)	0.8583 (0.0566)
Total EI	F1	0.4723 (0.0200)	0.6894 (0.0265)	0.5251 (0.0244)	0.7754 (0.0207)
	Acc.	0.4910 (0.0319)	0.6971 (0.0203)	0.5291 (0.0280)	0.7829 (0.0206)
	ROC-AUC	0.4868 (0.0395)	0.7537 (0.0154)	0.5764 (0.0166)	0.8990 (0.0147)
	PR-AUC	0.5787 (0.0372)	0.6565 (0.0298)	0.6179 (0.0159)	0.8802 (0.0132)

**Table 6:** Statistical comparisons between training groups for alternative backbone models using DeLong tests with repetition-level aggregation and binomial GEE analyses.

Backbone		High EI vs.	DeLong Test			Binomial GEE		
			Pooled ΔAUC	95% CI	q (FDR)	Odds Ratio	95% CI	q (FDR)
ReNet50	arousal	Low EI	−0.0107	[−0.0170, −0.0045]	<0.001	1.1642	[1.1155, 1.2149]	<0.001
		Total EI	0.0067	[0.0018, 0.0116]	<0.001	1.0815	[1.0452, 1.1190]	<0.001
	valence	Low EI	0.0043	[−0.0003, 0.0088]	<0.001	0.9053	[0.8663, 0.9460]	<0.001
		Total EI	0.0141	[0.0104, 0.0177]	<0.001	1.0167	[0.9860, 1.0483]	0.290
VGG16	arousal	Low EI	0.0059	[0.0021, 0.0097]	<0.001	0.8915	[0.8593, 0.9248]	<0.001
		Total EI	−0.0007	[−0.0043, 0.0029]	<0.001	0.9174	[0.8867, 0.9491]	<0.001
	valence	Low EI	0.0037	[0.0008, 0.0065]	<0.001	0.8424	[0.8009, 0.8861]	<0.001
		Total EI	0.0085	[0.0068, 0.0101]	<0.001	0.8643	[0.8266, 0.9036	<0.001

## Data Availability

The datasets presented in this article are not available because we jointly own the data with our partner organization, ETRI.
